# Health implications of dog-worn equipment: a review of known and alleged physical risks

**DOI:** 10.3389/fvets.2026.1711781

**Published:** 2026-05-12

**Authors:** Marcella Ridgway

**Affiliations:** College of Veterinary Medicine, University of Illinois Urbana–Champaign, Urbana, IL, United States

**Keywords:** canine health, collar, dog-worn equipment, harness, leash, muzzle, vest

## Abstract

Dogs are routinely managed using collars, harnesses and other equipment following practices that have been adhered to for centuries, yet concerns about potential adverse health effects have rarely been addressed through structured investigation. This review presents the existing literature regarding impacts of commonly-used dog-worn equipment, generally limited to studies of generated pressures and leash forces, effects on gait and intraocular pressure and a few case reports of equipment-related trauma. Additional studies are needed to specifically evaluate health risks and assess the validity of claimed adverse impacts of collars and other equipment. Until such evidence is established, heightened awareness of the associations of equipment with important and potentially vulnerable anatomic structures and functions as well as known risks presented herein can serve as a touchstone for safe, thoughtful practice in use of dog-worn equipment by all those who work with dogs.

## Introduction

Anyone who interacts directly with dogs, from owners, handlers and trainers to veterinary and other animal care professionals to animal control and shelter workers and volunteers, will likely engage in use of equipment which is worn by or applied to dogs. Many different types of equipment are utilized on dogs according to specific needs for control or the type of sport or work the dogs do and may include very specialized equipment as well as typical collar and harness restraints. Humans have applied equipment to dogs for more than 8,000 years (1). Despite the long history and frequent use of dog-worn gear, minimal structured investigation of its applications, benefits, risks and best-use practices has occurred. Much of its use is based on tradition, individual experience and anecdotal evidence to predict best practices, effectiveness and health impacts.

Equipment is placed on dogs for the expected benefits it may provide: for enhancement of health and safety of the dog, handler, other animals and general public achieved by restraint, control, and identification; for physical protection of the dog; for facilitation of training; and for expanding the dog’s capabilities (e.g., dog-worn camera, dog backpack/saddle bag). While dog-worn equipment is applied to the dog for a distinct purpose, its potential negative effects must always be taken into consideration even though controlled studies to define those risks are usually lacking. In the author’s opinion, equipment placed on and worn by dogs always has an impact on the dog, whether intentional or unintentional. This impact may be physical (e.g., skin sensation from contact with the equipment, leashed collar or harness restricting forward motion, equipment adding to the weight that the dog carries) but may also be mental (e.g., puppy scratching at newly-introduced collar). Equipment that impairs circulation or breathing may cause exercise intolerance or collapse and may predispose to heat stroke. Equipment may snag, trapping the dog in unsafe circumstances, or may chafe or trap irritants against the dog’s skin. The potential for equipment to injure or alter the function of underlying anatomic structures or to otherwise threaten the dog’s health, which may vary depending on how the equipment is being used in particular circumstances, must always be addressed and mitigated as possible. These considerations are of particular importance for working dogs, which are often subject to use of various types of dog-worn equipment for longer periods of time, potentially under more intense applications and challenging circumstances and, therefore, potentially with greater risk of negative impacts than are non-working dogs. Unfortunately, the information needed for evidence-based decisions regarding the use of dog equipment is usually absent. The purpose of this review is to inform best practices in use of dog-worn equipment by promoting awareness of body structures associated with and potentially impacted by dog-worn equipment; review the current literature on the physical impacts of dog-worn equipment; and, where potential impacts are presumed but unverified, disclose where further investigation is needed to help canine professionals make the best choices for safe and effective equipment use.

### Muzzles

Muzzles were developed to prevent dogs from biting and were first noted in ancient Persian religious writings (Aresta) circa 7th-6th century BC (2). Various constructs of muzzles evolved over time but it wasn’t until the beginning of the 13th century that features serving the safety and comfort of the dog (ability to eat and drink) were formally considered (3). Laws mandating muzzle use date to the late 19th or early 20th century, established primarily to prevent spread of rabies. Modern muzzles are available in a wide array of materials (leather, nylon, metal mesh, hard plastic, polyurethane, silicon) and styles. In addition to being used to prevent a dog from biting humans or other animals, muzzles are also used to protect the health of the muzzled dog. This includes preventing ingestion of foreign objects or potentially harmful substances (e.g., food-allergic dog on a neighborhood walk). Muzzles are used medically to prevent self-mutilation, maintain necessary fasting, prevent removal of bandages and indwelling medical devices (catheters, feeding tubes) and to provide therapeutic support to the jaw for jaw fractures or trigeminal neuritis (“dropped jaw”) patients where muzzles constructed of bandage tape are used during the recovery period (4–6).

Anatomic structures of the dog that are impacted by muzzles, and therefore susceptible to physical injury, include external surfaces of the head (skin of the face and jaws, nose leather, periorbital tissues and eyelids, eye globe) and potentially the proximal cervical region (most muzzles attach caudally by a collar-like strap that encircles the neck or by lateral straps that connect behind the dog’s ears). In addition to potential chafing or transmission of concussive forces to underlying structures, muzzles affect the ability of the dog eat, drink, sniff, and ‘signal’ physical behavioral cues (e.g., facial expression, calming signals) (7–9). Muzzles may accumulate liquids or detritus underneath that are then held as irritants against the skin and may be taken up into the mouth or nasal cavities. The most significant health risk that muzzles pose to dogs is by predisposing to heat stroke by restricting air flow and ability to pant. Dogs rely on panting and evaporative heat loss through the respiratory system for cooling the body (10–15). In response to heat stress, dogs are observed to breathe with more rapid, shallow breaths (panting). They open their mouths more widely and their exposed tongue lengthens. Muzzles have the potential to prevent these critical alterations that enhance heat dissipation and help to prevent heat injury. Predisposing dogs to life-threatening heat stress by impairing respiratory evaporative heat loss is the most significant health risk associated with muzzles: use of basket-type muzzles that do not restrict air flow and allow the dog to extend the tongue or open-mouth pant are believed to help mitigate this risk. Use of muzzles that restrict the ability of the dog to drink can predispose to dehydration, which further predisposes the dog to development of heat stress (16, 17). It is generally advisable to use muzzles for as short a period of time as possible, especially under warm, humid environmental conditions or periods of exertion, and to opt for muzzle styles that allow maximum air flow and ability of the dog to open the mouth into a wide smile and extend the tongue. Limiting the amount of time a dog is muzzled is especially important when more restrictive muzzles that hold the mouth tightly closed are used, such as to prevent biting during a nail trim or medical procedure. A pertinent example would be a working dog that is injured during exercise that must be muzzled to have an injury addressed: the dog’s need to pant to avoid exercise-induced heat injury must be recognized.

Arhant et al. (18) surveyed dog owners in Europe (predominantly Austria and Germany) about muzzle use: of 1862 respondents, 78.4% reported using a muzzle and 12.9% reported health problems in their dogs related to muzzle use, including physical injury to the skin or hair coat, eye injury, or problems with thermoregulation. Activities such as muzzle fighting may result in pinched skin edges, trauma to eyelids, eyes and teeth, and concussive forces placed through the nasal cavities with unknown consequences over time.

Another quite different risk associated with muzzles is overestimating their effectiveness in bite prevention. Dogs wearing muzzles to prevent biting may still cause bite injury even when the muzzle is properly fitted and secured and this risk appears to differ across muzzle types. Chvala-Mannsberger et al. (2) evaluated muzzle safety via online survey of 149 individuals including 110 dog trainers and 13 veterinarians in Austria and Germany in 2022. In that period, there were 63 reported bite injuries by muzzled dogs and severity of injury varied by muzzle type: for BioThane® (polyurethane) or hard plastic muzzles, over 60% of bites led to skin and tissue perforation while less than 50% of bites with leather muzzles resulted in tissue perforation. Metal muzzles generally caused impact injuries.

### Collars

Collars are used to control a dog’s mobility and to secure and carry information (ID, contact information, microchip and registration status) and specialized equipment (cameras, electronic devices): collars provide a secure attachment point that the dog cannot reach to remove by chewing. Collars have been in use for thousands of years: dogs wearing distinct recognizable collars were evident in ancient Egyptian art by the time of the Nagada II period 3,500–3,000 BC, and, by 1,570–1,069 BC, collars had become ornate and even recorded the dog’s name. Use of collars spread in Greece, where choke (unlimited slip) chain collars purportedly were developed (19), and in Rome: use of dog collars developed independently in other cultures worldwide in parallel with the importance of dogs in those cultures (19). Collars remain in wide use across the globe with many available styles and materials, all encircling the cervical (neck) region and serving primarily to secure and control the dog. The ventral neck of the dog is home to multiple vital anatomic structures: carotid arteries, jugular veins, vagosympathetic nerve trunk, recurrent laryngeal nerves, larynx, thyroid and parathyroid glands, and trachea (20–22). These critical tissues are minimally protected by overlying tissues but somewhat protected by a recessed position alongside the trachea in the jugular groove (20–22). They are further safeguarded by a dog’s ability to ventroflex the neck, bringing the chin toward the neck to cover the ventral neck region, with the cervical musculature providing dorsal and lateral protection (23). Collars completely encircle the dog’s neck and overlay the vulnerable ventral structures, thereby foiling normal protective mechanisms: ventroflexion (tucking the chin to the ventral neck) will not protect against circumferential collar pressure. The neck structures can withstand moderate pressure as major nerve and vessel trunks are recessed in the jugular groove and the trachea resists pressure due to rigid cartilage tracheal rings. The dog’s neck is most susceptible at the most cranial neck region, or ‘throat latch’, where overlying muscle, skin and hair coat are lighter and structures are more exposed.

Potential injury from use of dog collars is a focus of much well-warranted concern and also much conjecture and accusation, often with minimal supporting evidence: collars have received very little attention from the standpoint of structured studies despite the long history of collar use. Collar-related injuries, such as embedded collars, hanging, and limb or jaw trauma from collar entrapment, are well-known clinical entities that have received little to no attention in the literature beyond occasional case reports. Papageorgiou et al. (24) documented a case of traumatic tracheostomy in a dog that had been wearing looped wire as a collar: the dog was successfully treated with surgical repair. Pomorski (25) reported a case of contact dermatitis that was attributed to the dog’s leather collar. Grohmann et al. (26) described progressive neurologic dysfunction in a German shepherd following a 60-s period of strangulation by a choke collar during training: signs of disorientation, ataxia, circling, variable nystagmus, bilateral mydriasis, blindness and left-sided facial paralysis were attributed to brain hypoxia and edema secondary to brain ischemia from strangulation. Negative-pressure pulmonary edema (NPPE) is pulmonary edema that develops secondary to choking and associated forceful inspiratory efforts with an obstructed airway (27). In a retrospective study of 35 dogs with NPPE (28), 10 (28.6%) of the dogs had developed NPPE following a leash pull event. Six dogs experienced near-hanging (2 because of being trapped on the opposite side of an elevator door; 3 experienced near-hanging on a grooming table, 1 had the head entrapped between fence panels). Twenty-eight dogs (80%) survived to discharge after varying therapeutic interventions were employed. There was no follow-up to assess for long-term complications in these dogs.

Hanging injury and pathophysiology of hanging death has been assessed in humans (suicide, murder, execution) (29, 30): factors contributing to hanging injury or death include: asphyxiation (due to injury to larynx, hyoid, trachea (29, 31) causing airway obstruction), pharyngeal obstruction by upward displacement of the tongue, pulmonary edema (31), brain hypoxia (due to airway obstruction, carotid artery obstruction by compression), brain edema (due to jugular vein obstruction by compression), cervical spinal fracture (29, 31) with spinal cord injury (especially fracture of the dens from cervical hyperextension), and cardiac arrhythmia or arrest (due to hypoxia, vagal injury, carotid–cardiac baroreflex response to carotid compression) (31–33). Carotid artery injury from compressive forces or neck hyperextension with subsequent thrombosis and/or rupture leading to brain hypoxia has been reported as a cause of delayed hanging death (30). Published reports of hanging death in dogs include results of laboratory investigations in which dogs were used as animal models of asphyxiation (34–39) and limited information about hanging cases in the field (23, 28). However, there is adequate data to show that pathophysiology of hanging death in dogs has many parallels to that in humans as well as some variations: due to heavier muscling of the canine neck and differences in which blood vessels are most important for circulation of blood, and hence oxygen, to the brain, dogs remain conscious and show respiratory movements for a longer period during strangulation than humans (23). The ultimate cause of death is the same (brain hypoxia due to asphyxiation and/or impaired circulation) but onset of death is later (39).

Complete suspension of the body off of the ground is not necessary to cause hanging pathology and death. In two published case series of taped human hangings (suicides, autoerotic accidents), a majority (18/22) of hanging deaths involved partial suspension (body partially supported by feet or knees on the ground or prone body position) rather than complete suspension (limbs fully suspended with all body weight on the ligature): times to cessation of breathing and loss of consciousness did not differ significantly between complete or partial suspension (40, 41). Though parallel studies in dogs are lacking, this evidence in humans as well as documented pathophysiology of hanging injury in humans and dogs argues strongly against the practice of forceful lifting of the dog’s forequarters off the ground by the neck or collar or any strangulating use of a collar. All collars may be associated with strangulation injury, with the proximal ventral neck/laryngeal region recognized as the most susceptible to injury (42): the unlimited-slip or “choke” style collars are believed to pose the greatest risk because of their circumferential and unrestricted tightening and were associated with reported near-hanging of a dog during training (26).

Whether there are measurable health impacts in dogs of repeated lower-level episodes of collar pressure is unknown, though many postulate that collar injury may lead to thyroid dysfunction, tracheal damage, spine injury and other abnormalities in regional tissues (43–48). Documentation of any incidents or controlled investigations of these claims is, in almost all cases, absent. There is no reported documentation of tracheal trauma from chronic use of a collar for training despite claims to the contrary. Brinkman (42) studied experimental strangulation in dogs and found that it was very difficult to completely occlude the dogs’ trachea even using a garroting technique or tightening lever device but did not measure the forces being applied. In a radiographic study of tracheal collapse in small breed dogs, Beltran et al. (49) concluded that a normal canine trachea can withstand a direct compressive force of 20 mmHg without closure of the lumen while, in dogs with pre-existing tracheal collapse, 20 mmHg collar pressure severely compromised the tracheal lumen.

Embedded collars are a commonly encountered though under-reported health problem related to dog collars in which injury to cutaneous and sometimes deeper neck tissues is caused by collars that are left chronically in place and/or with improperly tight fit: underlying tissues can become inflamed to ulcerated to necrotic as a result of pressure, friction and secondary infection (24, 26, 50). Embedded collars are often related to circumstances where a dog grew without having its collar resized, in dogs with long dense coats where early signs of inflammation were not obvious, or in chronically-collared dogs that did not receive attentive care. Collar fit needs to be adjusted for weight changes, growth, and hair coat changes. While hanging injury and embedded collars are well-recognized clinical entities, there is no documentation within the scientific literature that intermittent pressure from leash tension on a collar results in direct tissue injury.

Intentional application of pressure to the neck to is used medically to treat some cardiac arrhythmias. Termed carotid sinus massage (CSM), the procedure is a form of vagal maneuver in which gentle sustained digital pressure is applied externally over the carotid sinus (at the bifurcation of the carotid artery). This leads to stimulation of the vagus nerve, which causes a decrease in heart rate and blood pressure and can be effective in treating supraventricular tachycardia in humans and dogs (51, 52). The vagus nerve also plays a role in control of breathing rate and depth (53). Whether pressure from routine collar use causes alterations in circulatory and respiratory functions by triggering a vagal reflex, as seen with CSM and as a component of hanging and near-hanging events, has not been reported.

### Harnesses and vests

Harnesses and vests are used to control a dog’s activity, carry attached items (identification, small packs, electronic devices), protect underlying body structures, provide specialized functions such as cooling or improving visibility of the dog, and serve as an attachment for objects to be pulled (sleds, skijoring or tracking/trailing handlers). Of commonly used equipment, harnesses and vests cover the largest surface area of the dog and have the greatest variability of design and fit and therefore the greatest variability of potential impact on the dog. The choice of harness style, material and location of attachment points depends on the intended use (e.g., routine leash-walking, pulling a sled, tracking/trailing), the best fit for an individual dog, and the preference of the handler (e.g., for how easy the harness is to place on the dog). Harnesses contact the neck, thorax and forelimb and, depending on design, may run across the thoracic inlet and distal cervical trachea, the shoulder or shoulder joint, and, significantly, the relatively-exposed shoulder extensor muscles (supraspinatus, biceps brachii) and their tendons. Except for some sled dog and skijoring harnesses, all have a continuous strap or panel that encircles the thorax and could be expected to restrict thoracic excursion (chest expansion) during inspiration. The greater the dog’s exertion, the greater the excursion of the thorax during the respiratory cycle to sustain ventilation and the greater the potential for a fixed harness strap to impair breathing functions. The impact of restricted thoracic excursion has been extensively investigated in humans: decreased chest wall expansion caused by constraining thoracic straps, vests or other external devices has consistently been shown to impair respiratory function and endurance in healthy adults (54–60). Such investigations in dogs are lacking but extrapolation from human studies seems reasonable in the absence of canine-specific data. Tactical harnesses and harnesses with a solid lateral chest panel connecting multiple thoracic straps would be suspected to restrict breathing more severely than harnesses with single or multiple independent thoracic straps. A thoracic strap that sits more cranially on the thorax (in the axillary region) may be advantageous from a breathing standpoint since the cranial thorax does not widen as much with respiration (ribs 1–9 are less mobile, being fixed at both rib ends [to vertebrae and sternebrae]) so cranially-situated straps would be expected to cause less restriction of thoracic excursion at times of maximal breathing effort (61, 62). Harnesses may also impact breathing by compressing the trachea but the impact via limiting thoracic expansion is the more significant.

Skin chafing by harnesses may occur because of problems with harness fit or irregularities (wrinkles, seams) in the equipment’s surface or if foreign material becomes trapped between equipment and the dog’s skin. Equipment that is lined with fleece, felt, neoprene or other padding would likely collect and hold debris more readily.

Published studies of dog harnesses focus on evaluation of their impact on the dog’s gait, on pressure distribution under the harness, and, infrequently, on leash tension in harness-walked dogs. These factors may be variably affected by harness design including attachment type and location and by position of the handler that is providing the resisting force (63–66). Harnesses are available in a wide variety of component materials and individual styles, all of which fall into one of two broader categories, labelled ‘restrictive’ or ‘non-restrictive’, based on whether or not they have a strap or other harness component which crosses the shoulder and/or front leg. Restrictive harnesses have a connection, usually a strap, which passes horizontally across the front of the dog’s chest (ventral neck at the thoracic inlet or prosternum) to connect on each side to a chest strap that encircles the thorax and, in doing so, runs across the scapula, shoulder blade, shoulder joint or upper arm (humerus). Non-restrictive harnesses do not cross the shoulder/forelimb and instead cross anterior to the shoulder and foreleg by a Y-shaped chest piece.

Use of the terms ‘restrictive’ and ‘non-restrictive’ stems from the assumption that a harness with a horizontal chest component crossing the forelimb (restrictive harness) would restrict motion of the forelimb and non-restrictive harnesses would not interfere. However, in all reported studies of harnesses and canine gait, all investigated harness types have impacted gait. In a comparative study of the effects of restrictive and non-restrictive harnesses on shoulder extension in dogs, both types of harnesses significantly reduced shoulder extension at a walk and at a trot; non-restrictive harnesses actually restricted shoulder extension more than restrictive harnesses at both gaits (67). In another study of the effects of harnesses and harness design on gait, Williams et al. (68) evaluated 66 dogs of varying breeds and found differences in stride length, weight distribution to the forelimbs, and minimum-maximum angles of the shoulder and elbow joints related to harness use during loose-leash walking. The presence or absence of gait changes, the magnitude of the changes and whether the changes were present or more severe in a restrictive versus non-restrictive harness varied across different breeds, highlighting the need to individualize the choice of harness type. In a pilot study of a detailed gait analysis method, Palya et al. (69) tested 2–4 different harnesses (both restrictive and non-restrictive) in 4 unleashed dogs walking on a treadmill: most gait parameters were significantly impacted by wearing a harness and which harness type was associated with less alteration in gait parameters varied with the individual dog, consistent with findings of a previous study (67). The gait alterations seen when dogs are wearing harnesses are evident without applied leash pressure (68, 69) and may persist when the harness is removed if dogs are walked in a harness frequently enough to develop muscle memory (70).

Guide dog harnesses, which are typically worn for protracted periods of time on an every-day basis, have been studied to identify harness characteristics which minimize alterations of normal movement. In these harnesses, it appears that the type of handle and position of attachment on the harness have greater impact on thoracic limb movement and stride length than whether a restrictive or non-restrictive harness is used (63, 71). Peham et al. (64) evaluated three types of guide dog harnesses and found that pressure distribution was asymmetric, with pressure being highest in the right sternal region with all harnesses, and that the right and left sternal region were almost constantly under a pressure load: surprisingly, the back region had minimal pressure loading. Differences across harness types were evident.

Tactical harnesses (sometimes called ‘service vests’) (72) are a type of harness that may be restrictive or non-restrictive. They are generally constructed of durable heavy canvas and nylon webbing, often with solid side panels and attachment points for badges and specialized equipment, and are commonly used on law enforcement, military and service (assistance) dogs. Foutz and Budsberg (72) evaluated truncal motion at a walk and trot in unleashed dogs wearing two different types of service vests (tactical harnesses). Dogs showed significant reduction of trunk motion and measurable gait differences when the vests were worn compared to equipment-free walking/trotting on a treadmill. Additionally, custom vests (solid construct encircling the dog’s trunk) were associated with greater reduction in truncal motion than adjustable harnesses (two truncal straps but open sides). A subsequent study (73) showed that wearing a tactical vest resulted in changes in kinematics in all three thoracic limb joints including shoulder abduction and reduced flexion at a walk, increased elbow extension and abduction at a walk and trot and increased carpal abduction and internal rotation at a walk. Overall, factors of harness weight, material or surface area coverage have not been evaluated for possible contributions to gait changes. The potential for these gait alterations to predispose the dog to musculoskeletal problems, although suspected, has not been documented. No investigations of gait impacts of ballistic vests or cooling vests have been reported.

Cooling vests have been evaluated for efficacy in mitigating overheating in exercising dogs. A phase-change (cooling packs) cooling vest and an evaporative cooling vest were compared to no cooling vest in a crossover study in exercised military working dogs (74). Mean body temperature measured immediately following exercise and at 15 min post-exercise were slightly lower in dogs wearing any cooling vest than in dogs with no vest. The evaporative vest performed better than the phase change vest based on lower mean temperatures at end exercise and 15 min post-exercise.

### Leashes

Leashes appear to be the first type of equipment used on dogs: stone drawings at Shuwaymis (9,000 + years BCE) (1) and Bhimbetka (7,000 BCE) show dog-like animals connected to human figures by a flexible-appearing line though no detail of how the line is secured to the dog is discernible. It seems feasible that collars may have originated as a loop of leash encircling the neck before becoming a separate entity. All-in-one leashes that have a neck loop as an intrinsic component of the leash remain in use today. Leashes serve to physically limit the area, direction and speed of a dog’s movement by tethering the animal to a handler or fixed point and are manufactured in a wide range of lengths and materials to suit various uses and handler preferences for style, comfort and ease of handling. Except for all-in-one leashes, where the collar loop has the same impacts as a collar, properly used leashes do not directly contact the body, instead exerting their physical impact on the dog indirectly through the collar or harness to which they attach. Dogs may, however, develop distinct leash aversions if the attachment process or presence of the leash is associated with a negative experience and may also show aggression toward the leash out of frustration of having their independent movement restricted (75, 76).

Published studies of leashes focus on the amount of tension developed in the leash for leash-walked dogs, inspired by concerns for animal welfare (77) and handler safety (pulling). Leash tension and associated pressures on the dog is the one area of the physical impact of dog-worn equipment that has received significant research attention (66, 77–83). These studies by their nature involve concurrent use of collars and/or harnesses. Leash tension may be impacted by many factors including the dog’s age, height, weight, conformation, health status, personality type and level of training and the handler’s height, mobility, stride length, hand-leash position, and handling skill level. Studies of leash tension must carefully consider and account for these influences and readers of these reports should carefully review study design before accepting study results and interpretations. The potential impacts of leash material [e.g., leather, nylon webbing, braided nylon, polyurethane (BioThane®), chain], width, thickness, weight, length, attachment device [e.g., various types of snaps, carabiners] or incorporation of a bungee segment have not been investigated in reported studies.

Methods used for measuring leash tension vary across studies. Shih et al. (65) developed a leash tension meter (load cell with data logging and transmitter communication functions) with a metal handle for the handler on one end and a leash attachment on the other to allow for real-time measurement of leash tension and pulling frequency. The meter was tested in dogs of varying sizes, ages and behaviors, showing higher leash tensions but lower pulling frequency in larger and heavier dogs and higher pulling frequency in young dogs. Dogs that were well-behaved exerted less leash tension but their handlers did not reciprocate with lower handler-generated leash tension. This meter was employed in an additional investigation of leash-pulling toward a desired reward (treat or toy) in dogs restrained by a flat collar versus back-attachment harness (78): dogs pulled harder (higher leash tension) and for a longer duration in a harness versus a collar for a food treat and there was no difference between collar and harness pulling for a toy treat; however, it was suspected that the dogs learned from the treat trial, which was first, and that this may have impacted the result for pulling toward a toy. Johnson and Wynne (79) evaluated leash pulling in shelter dogs using a strain gauge attached at the handler end of the leash and video recording of leash-walking sessions to compare pulling when using a martingale collar, metal prong collar, plastic prong-type collar or front-attachment harness. Dogs pulled significantly more on the martingale collar than the other three equipment types and tended to pull the least on the metal prong collar though differences in pulling with other equipment types were not significant. Carter et al. (80) employed a simulated canine neck model (plastic cylinder) and under-collar pressure sensors to evaluate differences in pressure exerted on the neck by a slip leash and seven different collar types at set levels of applied leash force. The overall pressure on the neck, the area over which the pressure was distributed, and the zone of the neck where the pressure was the highest varied significantly across collar types and the rope slip leash, with the rolled webbing collar showing the highest pressure on the neck at all leash force magnitudes. With increasing leash force, pressure under the collar became less evenly distributed for the padded webbing collar, the Lurcher-style collar, the rolled nylon collar and the stitched leather flat collar but pressure distribution remained consistent across leash force levels for the other styles. These results may not reflect collar impacts on the far more structurally complex live canine neck, on which skin and other structures can shift, muscle tone is dynamic and tissues may absorb some force through compressibility or ability to shift position.

Hunter et al. (81) utilized sensors placed between the collar and the ventral neck to examine collar pressure in leash-walked dogs wearing flat collars of the same size but constructed of 3 different materials. Significant differences in peak force and contact pressure related to differences in collar material were observed. Mean force also varied with direction of travel, being highest for counterclockwise direction and lowest for straight line walking. Bailey et al. (82) examined pulling forces in dogs wearing a flat collar versus a padded harness using a dynamometer secured between the handler end of the leash and a climbing harness worn by the handler: this gave the handler a hands-free role in an effort to minimize the handler contribution to variation in measured leash tension. Peak pulling force increased as the size of the dog increased; however, small dogs were found to pull more strongly than medium or large dogs when peak pulling force was determined as a percent of body weight. Dogs were found to pull with greater mean and peak force when wearing a back-clip harness compared to a collar. Thus, the only studies to objectively assess differences in pulling forces between collars and harnesses refute the widely held belief that dogs walked in a harness pull less than dogs walked in a collar and, in fact, support that dogs pull harder or at least as hard in a harness (78, 82). These studies do not provide insight into why this is the case, which might include differences in the angle of the leash between handler and dog, differences in surface area and anatomic structures impacted by the equipment, or potentially a dog’s intrinsic sensitivity to pressure on a particular body area, such as the ventral neck. Results may not apply across all equipment styles and types of materials as differences in pulling forces with collars of the same overall style constructed of different materials, as well as collars of different styles (flat buckle, unlimited slip/choke, martingale), have been reported (80, 81).

In evaluating factors which may impact veterinary diagnostic gait analysis in dogs, Keebaugh et al. (66) found that gait analysis in small-breed dogs may be affected by on which side the handler and leash are positioned: study dogs tended to shift weight away from the leash-handler side to the opposite forelimb, creating gait asymmetry, but hindlimb gait was not significantly impacted. In leash-walked large breed dogs assessed at slow and fast stepping speeds, Cotton et al. (83) found that dogs maintained the same stride length at different velocities, instead altering the duration of different phases of the stepping motion to effect speed changes. They postulated that this might relate to restriction of the dog by the handler’s stride length and that the handler may thus influence gait assessment. It is important to recognize that studies of equipment pressure and leash forces have assessed circumstances of routine leash-walking at a walk and trot and results may not apply to dogs under working conditions where the dogs’ heightened level of effort or ‘drive’ for their work might cause them to exert more pull and accept more countering pressure than during routine leash walking or gaiting on a treadmill. In addition to higher levels of drive, working dogs might have a different level of training, conditioning and lean body mass than the general dog population, elements that were not described or controlled in the existing body of evidence but that might affect leash tension results.

Leash walking has been associated with injury to humans including fractures, dislocations, amputations, strains/sprains, abrasions, and contusions, usually associated with tripping or entanglement resulting from strong pulling by the dog (54, 84). Forrester (85) reported a rate of leash-injury prompting an emergency department visit of 25.4 per 1,000,000 population in 2001 increasing to 105.5/1,000,000 in 2018. Injuries occurred most commonly at home (37.4%) followed by street/highway (12.8%) and other public property (12%). Ocular injury in a human caused by recoil of a retractable leash has been reported (86) and retractable leashes routinely carry a printed warning of amputation risk. No comparable reports of leash-related injuries exist for dogs although it is suspected that dogs could be subject to such injuries, especially those resulting from entanglement.

Leash-pulling on a collar has been suggested to cause or exacerbate a number of canine health conditions, most often with little to no documentation to support such claims, as logical as some might seem. Pauli et al. (87) documented significant increases in intraocular pressure (IOP) in dogs pulling against a collar but not a harness; the increase was deemed to be potentially deleterious to eye health, especially for dogs with glaucoma or weak/thin corneas. Bailey et al. (88) examined brachycephalic and dolichocephalic dogs at rest and with loose-leash walking in a collar and in a non-restrictive harness and found significant increases in IOP over baseline in brachycephalic dogs wearing a collar when stationary and when loose-leash walking. In dolichocephalic dogs, only exercise in a collar was associated with increased IOP. Harness use was not associated with IOP elevations, supporting their use in dogs with certain ocular diseases. Respiratory rate increased significantly with collar use at rest and with leash-walking, and with leash-walking in a harness in brachycephalic dogs: respiratory rate was not impacted in dolichocephalic dogs. Other claims of collar-related injury due to leash-pulling, such as damage to the thyroid gland, trachea, jugular veins, or carotid arteries, are not supported in the literature: there are no published reports documenting neck injuries resulting from leash-pulling though some include extrapolations claimed to represent evidence of cause [e.g., (77, 81)]. Accessing the references for such claims reveals the lack of actual evidence.

## Conclusion

There is a paucity of evidence regarding best designs and best practices for use of standard dog equipment and, while potential for harmful impacts on dogs’ health is suspected, little evidence exists to define the actual risks. Isolated case reports document severe trauma associated with misuse of collars. Multiple studies document that gait is affected by leash-walking with a collar or harness and that simply wearing a collar can be associated with increased intraocular pressure in some (brachycephalic) dogs. Whether there are other adverse physical effects of sustained use of dog-worn equipment remains unknown without additional study to replace assumption, extrapolation and conjecture with real evidence on which to base decisions about equipment use. Until additional structured investigation can verify actual health impacts, dispel myths, and inform best equipment design and safe use practices, consideration of the existing literature and awareness of basic canine anatomy and function that may be impacted by dog-worn equipment as presented here provides all who work with dogs with an important step forward in assuring safest equipment use practices.
